# Mitigative role of cysteamine against unilateral renal reperfusion injury in Wistar rats

**DOI:** 10.3389/fphar.2024.1456903

**Published:** 2024-09-10

**Authors:** Babatunde Adebola Alabi, Okot-Asi Nku-Ekpang, Sodiq Kolawole Lawal, Ezekiel Olugbenga Iwalewa, Temidayo Omobowale, Richard Ajike, Ridwan Abiodun Lawal

**Affiliations:** ^1^ Department of Pharmacology and Therapeutics, Bowen University, Iwo, Nigeria; ^2^ Department of Pharmacy, Kampala International University in Tanzania, Dar es Salaam, Tanzania; ^3^ Department of Physiology, University of Calabar, Calabar, Cross River, Nigeria; ^4^ Department of Anatomy, University of Botswana, Gaborone, Botswana; ^5^ Department of Pharmacology and Therapeutics, University of Ibadan, Ibadan, Oyo, Nigeria; ^6^ Department of Veterinary Medicine, University of Ibadan, Ibadan, Oyo, Nigeria; ^7^ Department of Physiology, Ladoke Akintola University of Technology, Ogbomosho, Oyo, Nigeria; ^8^ Department of Biochemistry, University of Lagos, Lagos, Nigeria

**Keywords:** renal injury, transcription factor, inflammatory mediators, caspase 3, kidney transplant

## Abstract

**Background:**

Ischemia-reperfusion injury (IRI) is unavoidable during kidney transplant and it is responsible for delayed or non-function after kidney transplantation. Cysteamine is the standard drug in the management of nephropathic cystinosis and its extra-renal complications. Thus, we designed this study to investigate its potential against renal reperfusion injury.

**Results:**

Significant elevation of H_2_O_2,_ MDA, and nitrite and reduced GPx, GSH, and protein thiol in the Ischemia-reperfusion injury rats was reversed by cysteamine (50 and 100 mg/kg). Serum MPO, TNF-α, IL-1β, creatinine, and AOPP were significantly elevated in IRI while rats treated with cysteamine revealed a significant decrease (*p* < 0.05) in the activities of these pro-inflammatory and renal injury markers.

**Conclusion:**

Based on its activity against inflammation, apoptosis, and free radical-induced stress, cysteamine has great potential to be used as a kidney transplant pre-operative drug to prevent renal reperfusion injury.

## Background

Chronic kidney disease (CKD) entails long-term damage to the kidneys and can lead to renal failure (end-stage kidney disease). To date, CKD is estimated to affect 10% world’s population and its prevalence keeps increasing due to the higher incidence of hypertension and diabetes worldwide (Kovesdy, 2022). Based on the global ranking list of chronic diseases causing death, CKD was ranked 12th in the year 2015 (Neuen et al., 2017).

For end-stage kidney disease (ESKD) patients, dialysis and kidney transplantation are major treatment options with the latter a better option than dialysis because it provides a sustainable and higher quality of life (Hole et al., 2020). Despite being a better treatment strategy for the management of ESKD, suitable kidney donors for ESKD patients are scanty. For instance, worldwide renal transplantation performed in the year 2018 was seventy-six (Mudiayi et al., 2022). The shortage of kidney donors has caused many transplant centers to accept large numbers of older and higher-risk organs recovered from deceased donors (Mohan et al., 2018). These are sub-optimal quality organs that can affect the outcome of transplantation negatively (Von Moos et al., 2020). Also, kidneys retrieved from brain death donors, and circulatory death donors have reduced the viability of kidneys used for transplantation (Sun et al., 2018), thereby compromising a successful immediate function and long-term graft survival after transplantation.

The risk of post-transplant primary organ loss of function, delayed graft function, and graft rejection is potentiated by surgical-associated ischemia/reperfusion injury (IRI). IRI is unavoidable during kidney transplant and it is responsible for delayed or non-function after kidney transplantation (Nieuwenhuijs-Moeke et al., 2020). Although the pathophysiology of IRI is complex, an increase in free radical production, activation of pro-inflammatory mediators, programmed cell death, transcriptional reprogramming, and immune system are classical pathways of reperfusion injury (Salvadori et al., 2015). IRI is associated with strong pro-inflammatory responses and short-term rejection due to the immunogenicity of humoral antibodies and T cell-mediated events (Ravindranath et al., 2021). To achieve the best possible post-transplant function, and prevent primary non-function, delayed graft function, and rejection, it is very crucial to minimize ischemia/reperfusion injury during a kidney transplant. New therapeutic targets that can attenuate IRI via intracellular pathways can optimize the condition of every graft-to-be.

Given the protective role of sulfhydryl-containing agents like N-acetylcysteine, N-acetylcysteine-amide, meso-2,3-dimercaptosuccinic acid, British anti-lewisite, D-penicillamine, amifostine and others (Pfaff et al., 2020) against apoptosis, DNA damage, inflammation, oxidative stress, and stress-induced transcription factors as reported in previous studies, we speculated that cysteamine has the potential to become a new drug target against IRI during a kidney transplant. Cysteamine is a sulfhydryl/aminothiol-containing drug approved by the FDA for the treatment of nephropathic cystinosis to reduce lysosome cysteine accumulation (Guha et al., 2019). Cysteamine was reported in previous studies to protect against testicular torsion/detorsion (Afolabi et al., 2021). In addition, Okamura et al. (2014a) also revealed that cysteamine can prevent chronic kidney injury via modulation of oxidative stress and inhibition of myofibroblast activity. In this study, we intend to investigate the mitigative role of cysteamine against renal reperfusion injury via attenuation of pro-inflammatory transcription factors, inflammatory cytokines, apoptosis oxidative stress, and renal disease biomarkers.

## Methods

### Experimental animal design and treatment

Male rats of Wistar strains with an approximate weight of 150-200 g, were given free access to pellet feed and water. We allowed the rats to acclimatize for 14 days before the commencement of the study. We obtained ethical approval from the University research ethics committee and the approval number designated for animal use was BUTH/REC-148.

### Reagents

TNF-α and IL-1β ELISA kits were purchased from Elabscience Biotechnology (14,780 Memorial Drive, Suite 216, Houston, Texas, USA). Also, apoptotic-related caspase 3 (Catalog E-AB-22128), and p65NFkB are products of Elabscience Biotechnology. Advanced oxidized protein products (AOPP) ELISA kit was purchased from MyBioSource Biotechnology Company, Southern California, San Diego, USA (Catalog MBS028634). Ketamine hydrochloride injection (50 mg/mL) was a product of Indiamart and xylazine injection (300 mg/mL) was purchased from NexGen Animal Health (NC-0588)

Twenty-eight male Wistar rats were divided into four groups (seven rats per group; n = 7) as follows:

50 mg/kg Cysteamine + IRI; rats were treated with 50 mg/kg cysteamine (i.p; intraperitoneal) for 48 h before renal reperfusion injury.

100 mg/kg Cysteamine + IRI; rats were treated with 100 mg/kg cysteamine (i.p; intraperitoneal)) for 48 h before renal reperfusion injury.

### Procedure for renal ischemia-reperfusion injury induction

The rats were weighed and anesthetized with 10 mg/kg of xylazine and 50 mg/kg of ketamine (intraperitoneal route). As Zheng et al. (2018) described, a midline incision was made at the abdominal region, and the right kidney was located. The right renal artery was spotted and clamped with small non-crushing forceps to induce renal ischemia and sutured with 2–0 chromic suture. After 30 min of ischemia, the rats were opened and the clamp was removed to induce renal reperfusion injury for 24 h.

### Serum and tissue collection

The rats were injected (intraperitoneal route) with a mild dose (25 mg/kg) of ketamine after 24 h of reperfusion injury for blood sample collection through the retro-orbital plexus. The rats were euthanized using isoflurane (5%) in a desiccator for kidney collection. The kidney was harvested, cleared of adherent tissue, and weighed. The harvested kidney was cut longitudinally such that, one section was submerged inside formaldehyde for histological assessment while the other section was homogenized for the assessment of biochemical parameters.

### Biochemical analysis

According to the modified protocols described by Afolabi et al. (2021), the tissue level of glutathione peroxidase, reduced glutathione, protein thiol, Superoxide dismutase, hydrogen peroxide, malondialdehyde, myeloperoxidase, and nitric oxide derivative was determined.

The levels of IL-β, TNF-alpha, and creatinine in the serum of rats were estimated with an ELISA kit, based on the protocol described by the manufacturer. Serum AOPP level was estimated using the MyBioSource ELISA kit.

### Tissue processing

By using a scalpel blade, a 4 mm piece of renal tissue was cut off and placed in a pre-labeled cassette and the microtome was set at 5 µm thickness to cut off the renal tissue for H and E. The tissue was further submerged in formal saline (10%) for 1 day of fixation. The tissues were later processed and stained with Haematoxylin and Eosin.

### Immunohistochemistry

Pro-apoptotic related caspase 3, and pro-inflammatory transcription factor p65NFkB expressions were evaluated using immunohistology, as described by Alabi et al. (2020). Briefly, the renal tissues were preserved with 10% formlaldehyde, and 5-μm thickness sections of the tissue were cut onto poly-lysine-coated glass slides. To retrieve the antigens, the sections were boiled in 10 mM citrate buffer (pH 6.5) for 20 min. Sections were incubated with hydrogen peroxide for 15 min to minimize non-specific staining and then rinsed three times (5 min each) with 1 × PBST (0.05% Tween 20). Blocking solution was applied for 10 min and then sections were incubated with elabscience diluted primary antibodies for caspase three and p65NFkB. Anti-NFkB (p65) (dilution 1:200), and anti-caspase 3 (1:200) were incubated overnight at 4 °C. Further processing was done using the Ultra Vision Plus Detection System Anti-Polyvalent, HRP/3,3′-diaminobenzidine (DAB) (Ready-To-Use) staining kit from Thermo Scientific system. The peroxidase complex was visualized with DAB. Last, the slides were counterstained with hematoxylin, cleaned in xylene, dehydrated with ethanol and then DPX mounting microscopic (Olympus BX-51) analysis was done. Quantitative analysis of protein expression from the photomicrograph taken was done through the ImageJ software package from Nottingham through the total area of positive staining.

### Statistical analysis

We represented data as mean ± standard error of mean and analysis of variance was used to compare within the groups and post-hoc test (Tukey test) for multiple comparisons. Graph Pad Prism software version five was used for analysis.

## Results

### Effect of cysteamine on creatinine and AOPP of IRI rats

**FIGURE 1:**
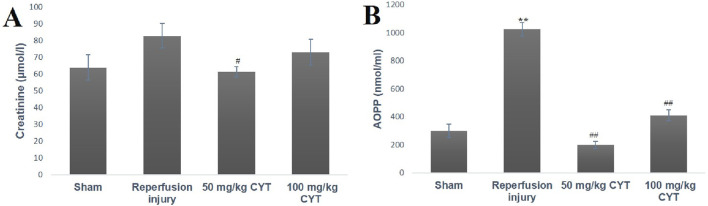
Bar chart expression of renal reperfusion injury and cysteamine effects on Creatinine **(A)** and AOPP **(B)**. Values were represented as mean ± SEM, for seven rats per group (n = 7). *Values differ significantly from sham control (*p < 0.05, **p < 0.01, ***p < 0.001). # Values differ significantly from renal reperfusion injury group (#*p* < 0.05, ##*p* < 0.01, ###*p* < 0.001).

The induction renal ischemia/reperfusion injury (IRI) increased the serum level of advanced oxidized protein products (AOPP) (*p* < 0.05) when compared with the sham control rats, although, there was no significant increase in the creatinine level (*p* > 0.05). The pretreatment of rats with cysteamine (50 mg/kg) before inducing the renal IRI rats reduced the serum level of creatinine and AOPP significantly (*p* < 0.05). The administration of 100 mg/kg cysteamine could not exert any significant effect on serum creatinine levels (Figures 1A, B).

### Effect of cysteamine on oxidative stress parameters of IRI rats

**FIGURE 2:**
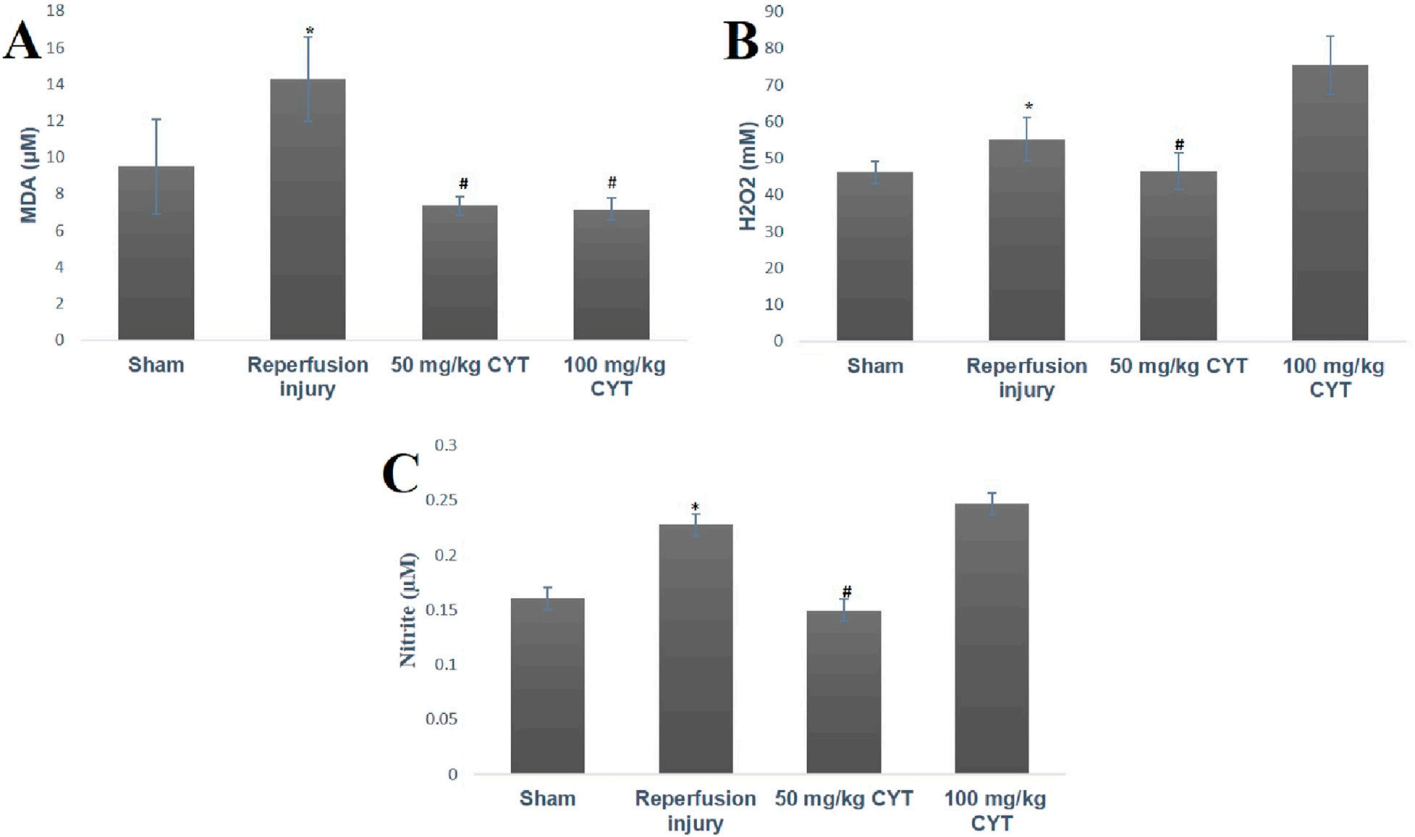
Bar chart expression of renal reperfusion injury and cysteamine effects on renal tissue level of MDA **(A)**, H_2_O_2,_ **(B)** and nitrite **(C)**. Values were represented as mean ± SEM, for seven rats per group (n = 7). *Values differ significantly from sham control (*p < 0.05, **p < 0.01, ***p < 0.001). # Values differ significantly from renal reperfusion injury group (#*p* < 0.05, ##*p* < 0.01, ###*p* < 0.001).

The tissue levels of oxidative stress markers (MDA, H_2_O_2,_ and nitrite) were elevated by the reperfusion injury. As illustrated in Figures 2A–C, the tissue levels of malondialdehyde (*p* < 0.05), hydrogen peroxide (*p* < 0.05), and nitrite (*p* < 0.01) were elevated in IRI rats compared with rats in the sham group. The lipid peroxidation biomarker (MDA) was significantly reduced (*p* < 0.05) in the cysteamine (50 mg/kg and100 mg/kg) treated IRI rats (Figures 2A–C) when compared to the IRI rats. Although the pretreatment of rats with 50 mg/kg cysteamine decreased the levels of (H_2_O_2,_ and nitrite) significantly when compared with IRI rats, the pretreatment of rats with 100 mg/kg cysteamine could not reduce the nitrite and hydrogen peroxide levels (Figures 2A–C).

### Effect of cysteamine on the antioxidant status of IRI rats

**FIGURE 3:**
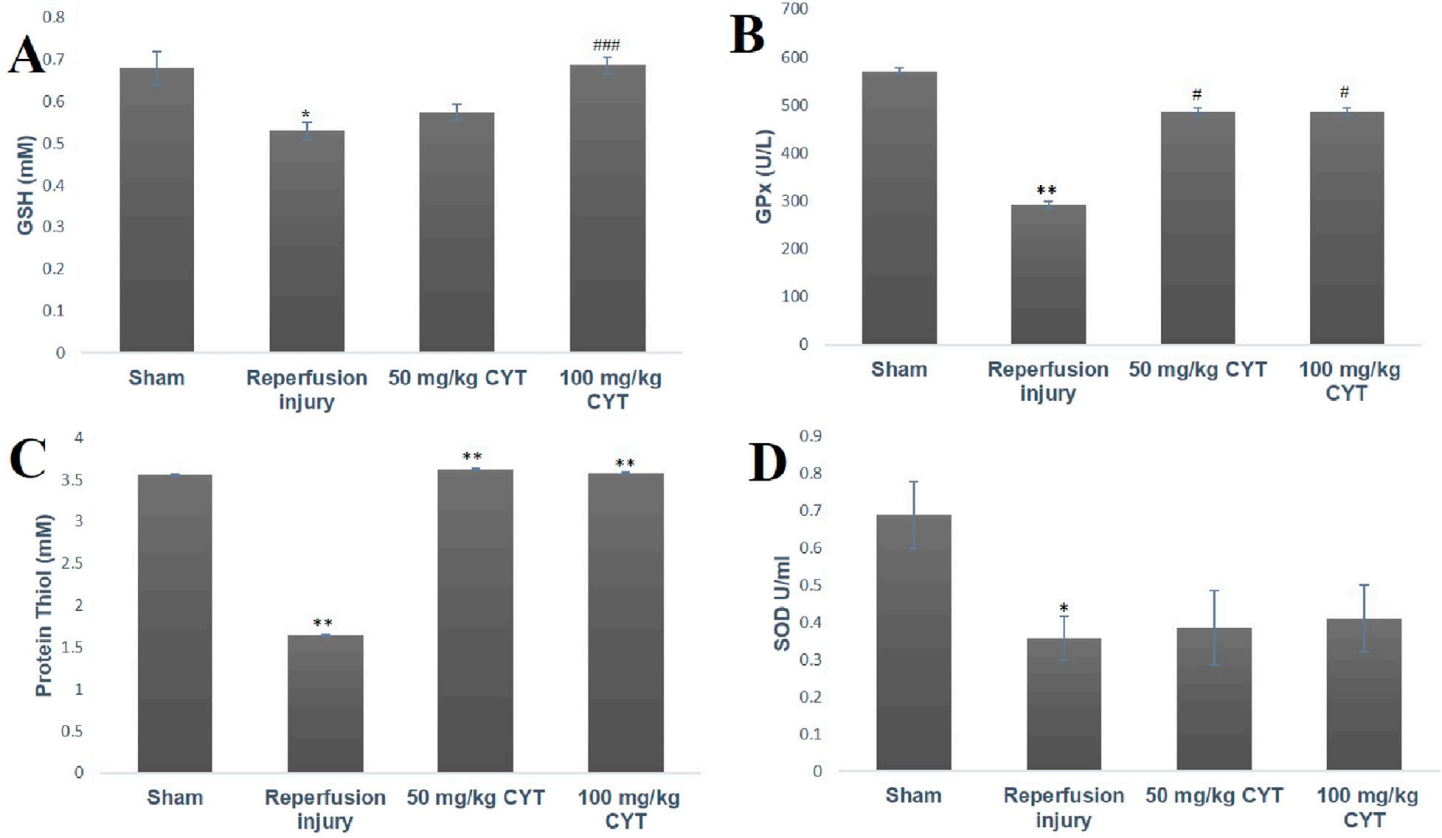
Bar chart expression of renal reperfusion injury and cysteamine effects on renal tissue level of GSH **(A)**, GPx **(B)** protein thiol **(C)**, and SOD **(D)**. Values were represented as mean ± SEM, for seven rats per group (n = 7). *Values differ significantly from sham control (*p < 0.05, **p < 0.01, ***p < 0.001). # Values differ significantly from renal reperfusion injury group (#*p* < 0.05, ##*p* < 0.01, ###*p* < 0.001)

The effect of IRI on the antioxidant levels of treated and sham control rats is represented in Figures 3A–D. The tissue levels of GSH, GPx, and protein thiol were significantly depleted in IRI rats (*p* < 0.05, *p* < 0.01 and *p* < 0.01) when compared with the rats in the sham group. The cysteamine-pretreated IRI rats showed a significant increase in the tissue level of GSH, GPx, and protein thiol (*p* < 0.001, *p* < 0.05, and *p* < 0.01) when compared with IRI rats. The superoxide dismutase (SOD) level was significantly reduced (*p* < 0.05) in the IRI group but cysteamine could not exert any significant effect on tissue SOD (Figures 3A–D).

### Effect of cysteamine on the inflammatory cytokines of IRI rats

**FIGURE 4:**
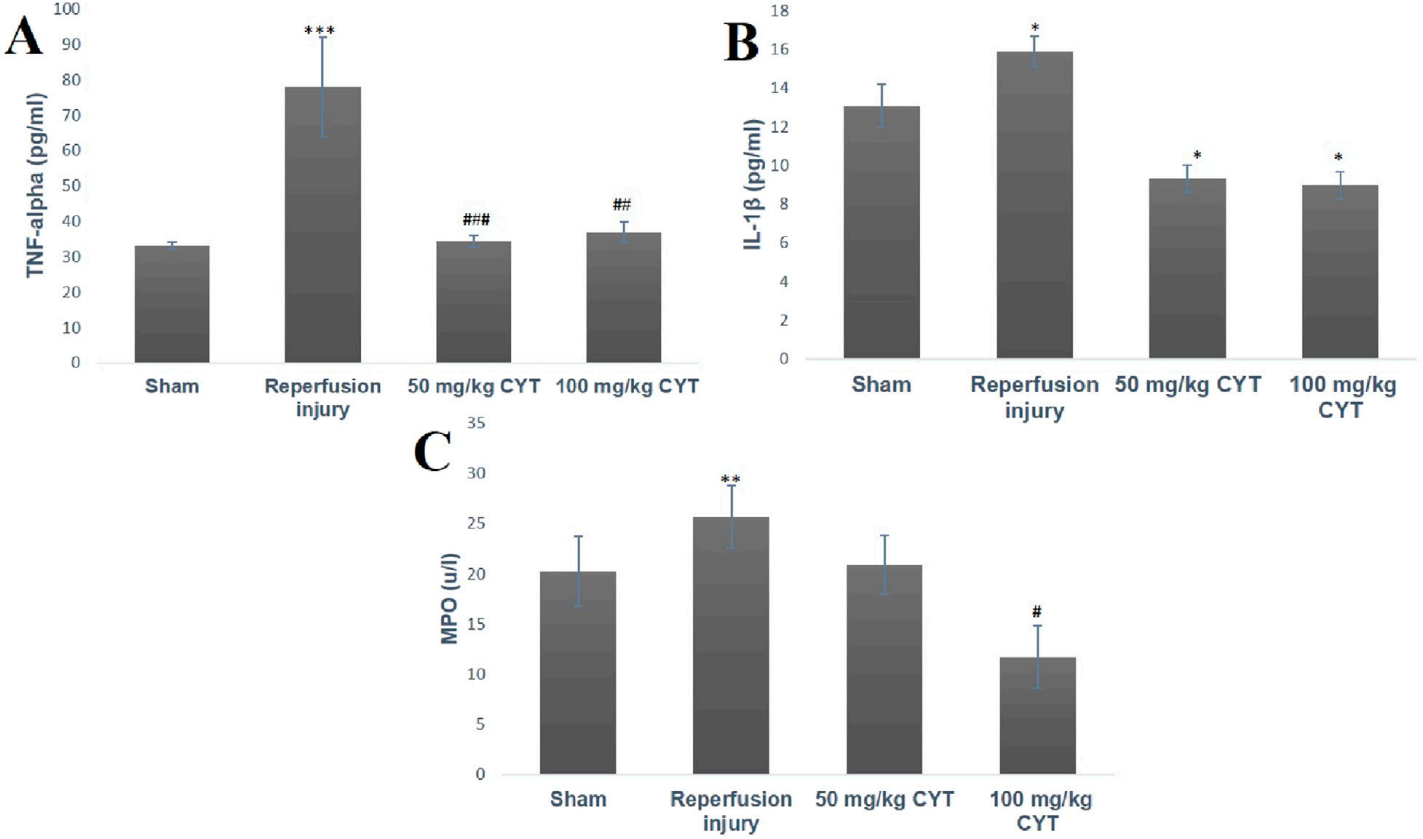
Bar chart expression of renal reperfusion injury and cysteamine effects on the serum level of TNF-α **(A)**, IL-1β **(B)**, and MPO **(C)**. Values were represented as mean ± SEM, for seven rats per group (n = 7). *Values differ significantly from sham control (*p < 0.05, **p < 0.01, ***p < 0.001). # Values differ significantly from renal reperfusion injury group (#*p* < 0.05, ##*p* < 0.01, ###*p* < 0.001)

When compared with the rats in the sham group, the serum levels of TNF-α (*p* < 0.001), IL-1β (*p* < 0.05), and MPO (*p* < 0.01) were elevated in the IRI rats (Figures 4A–C). The pretreatment rats with 50 mg/kg and 100 mg/kg cysteamine reduced the serum level of TNF-α (*p* < 0.001, *p* < 0.0001) when compared with the IRI group. The serum level of IL-1β was significantly reduced by 50 mg/kg and 100 mg/kg cysteamine treatment when compared with IRI rats without treatment (*p* < 0.05). Also, a significant reduction in the levels of MPO (*p* < 0.05) was observed in 100 mg/kg cysteamine rats when compared with the IRI group, but 50 mg/kg cysteamine reduced the MPO level insignificantly (Figures 4A–C).

### Effect of cysteamine on the tissue expression of apoptotic-related proteins

**FIGURE 5:**
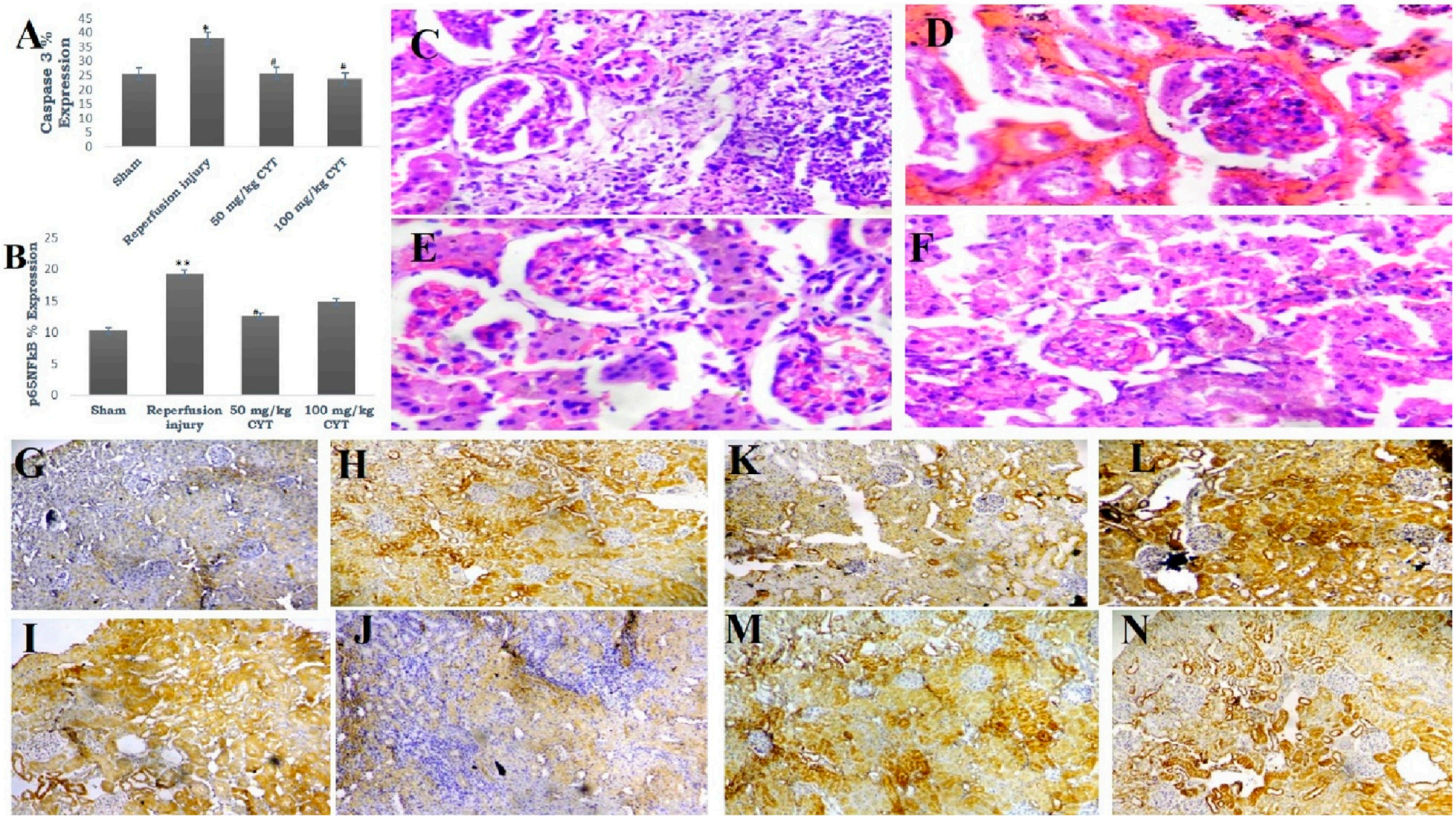
**(A, B)** Bar chart expression of renal reperfusion injury and cysteamine effects on caspase three and pro-inflammatory transcription factor. Values were represented as mean ± SEM, for seven rats per group (n = 7). *Values differ significantly from sham control (*p < 0.05, **p < 0.01, ***p < 0.001). # Values differ significantly from renal reperfusion injury group (#*p* < 0.05, ##*p* < 0.01, ###*p* < 0.001). **(C–F)** Photomicrograph of renal histoarchitecture showing the effect of reperfusion injury on rats in group C (sham). Rats in group D (reperfusion injury without treatment). Rats in groups **(E, F)** (50 and 100 mg/kg cysteamine) (mag. X 400). **(G, H, I and J)** Photomicrograph representation of renal tissue expression of caspase 3: rats in group G (sham) showed a mild expression of caspase 3; rats in group H (reperfusion injury without treatment) revealed severe expression of caspase 3; rats in groups **(I, J)** (50 and 100 mg/kg cysteamine) showed a moderate expression (mag. X100). **(K, L, M and N)** Photomicrograph representation of renal tissue expression of p65NFkB: rats in group **(K)** (sham) showed a mild expression of p65NFkB; rats in group **(L)** (reperfusion injury without treatment) revealed severe expression of p65NFkB; rats in groups **(M, N)** (50 and 100 mg/kg cysteamine) showed a moderate expression (mag. X100).

**FIGURE 6:**
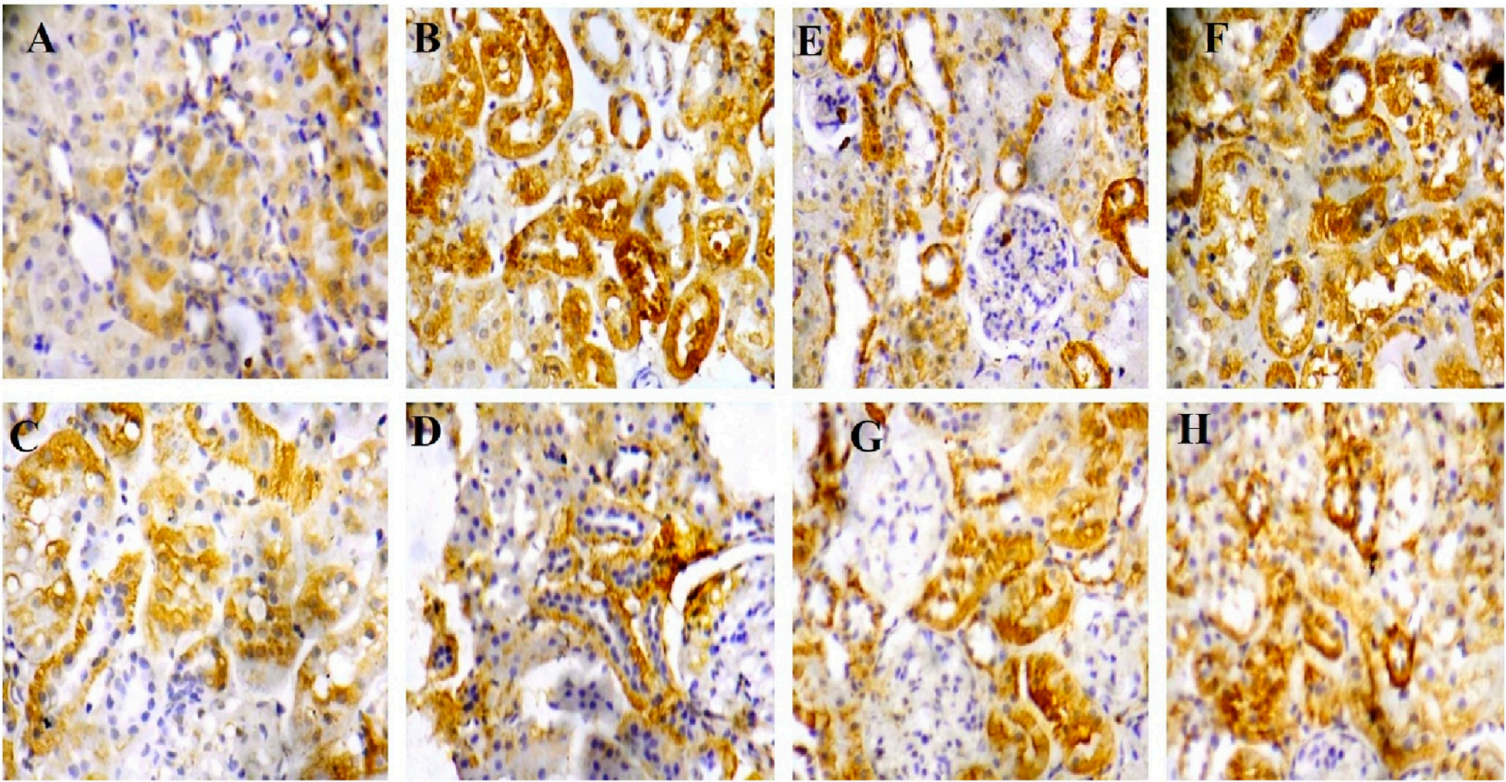
**(A–D)** Corresponding high-magnification image (mag. X400) of renal tissue expression of caspase 3: rats in group A (sham) showed a mild expression of caspase 3 (Figure 5G correspondence); rats in group B (reperfusion injury without treatment) revealed severe expression of caspase 3 (Figure 5H correspondence); rats in groups **(C, D)** (50 and 100 mg/kg cysteamine) showed a moderate expression (Figures 5I, J correspondence). **(E–H)** Corresponding high-magnification image (mag. X400) of renal tissue expression of p65NFkB: rats in group E (sham) showed a mild expression of p65NFkB (Figure 5K correspondence); rats in group F (reperfusion injury without treatment) revealed severe expression of p65NFkB (Figure 5L correspondence); rats in groups **(G, H)** (50 and 100 mg/kg cysteamine) showed a moderate expression (Figures 5M, N correspondence).

The renal tissue expression of apoptotic-related caspase three and Bax were significantly increased (*p* < 0.05) in IRI rats without treatment. The cysteamine plus IRI treatment rats (50 mg/kg and100 mg/kg) showed a significant decrease in tissue expression of caspase three (*p* < 0.05) compared with the sham control rats (Figures 5A, G, H, I, J and 6A–D).

### Effect of cysteamine on the tissue expression of proinflammatory transcription factor

Renal expression of p65NFkB was significantly elevated in IRI rats (*p* < 0.01) when compared with the sham group. The treatment of rats with 50 mg/kg (*p* < 0.01) reduced the renal tissue expression of p65NFkB compared with the IRI group. Treatment of rats with 100 mg/kg also reduced p65NFkB level (*p* = 0.05) (Figures 5B, K, L, M, N and 6E–H).

### Photomicrograph of the kidney section

The photomicrograph of the kidney section (sham control rats) showed normal architecture, and the renal cortex showed normal glomeruli with normal mesangial cells and capsular spaces. Few glomeruli appeared atrophic with compact glomerular elements, most of the renal tubules appeared normal, and the interstitial spaces showed a large area of infiltration of inflammatory cells (Figure 5C).

In IRI rats, a photomicrograph of the kidney section showed poor architecture, with the renal cortex showing glomeruli with congested capillaries and renal tubules appearing moderately normal. The interstitial spaces show severe hemorrhage (Figure 5D).

Photomicrograph representation of cysteamine (50 and 100 mg/kg) kidney sections revealed normal architecture, renal cortex showed normal glomeruli with normal mesangial cells and capsular space. The renal tubules showed normal and collapsed tubules lacking luminal spaces, and interstitial spaces appeared normal (Figures 5E, F).

## Discussion

Renal ischemia/reperfusion injury (IRI) cannot be avoided during surgical transplant and this can result in a non- or delayed functional kidney after transplant (Ponticelli, 2014; Salvadori et al., 2015). From the results obtained in this study, occlusion of the renal artery for 30 min followed by restoration of blood flow caused oxidative stress, as evidenced by an enhanced level of oxidative stress biomarkers and concomitant depletion of renal antioxidant status. Oxidative stress biomarkers such as MDA, nitrite, and H_2_O_2_ were significantly increased during reperfusion injury while renal tissue levels of GSH, GPx, and protein thiol were significantly reduced. Elevation of oxidative stress markers supports previous studies by Martin et al. (2019); Chouchani et al. (2016) and Afolabi et al. (2022) that showed a burst of ROS production during tissue reperfusion after the episode of ischemia. Excess production of free radical species after ischemia/reperfusion has been attributed to infiltration of tissue with neutrophils, and macrophages along with disruption of the mitochondrial electron transport chain. IRI-induced tissue antioxidant depletion observed in this study correlates with previous studies by Omobowale et al (2016); Alabi et al. (2022) and Afolabi et al. (2021) that reported a significant reduction in tissue antioxidant status during reperfusion injury. Pretreatment of rats with 50 mg/kg and 100 mg/kg cysteamine seems to enhance intracellular thiol antioxidant and antioxidant enzyme GPx, thereby protecting the renal tissue from reperfusion injury. GSH and protein thiol have been regarded as the most powerful antioxidant system in a cell (Wang et al., 2012). They are known to scavenge reactive oxygen and nitrogen species via redox reaction due to the presence of sulfhydryl moiety. They can pair electrons with free radicals directly or improve GPx activities (Dinkova-Kostova AT, 2012). As a sulfhydryl group-containing drug, cysteamine is known to penetrate the lysosome and split cysteine and cysteine-cys disulfide, leading to rapid depletion of cellular and tissue cytine via redox reaction (Chen et al., 2022). Most of the actions of cysteamine have been linked with the presence of thiol-disulfide exchanges with cysteine. The same thiol moiety has been shown to be responsible for the mopping of excess free radicals and enhancement of tissue reduced gluthathione level as observed in this study. The presence of cysteamine thiol (SH) attachment appears to be responsible for the depletion of lysosomal cysteine during cystinosis and its antioxidant effect against renal reperfusion injury.

Although cysteamine (50 mg/kg and 100 mg/kg) enhanced renal tissue antioxidant thiol, and reduced the lipid peroxidation (MDA) level in this study, the enhancing effects of 100 mg/kg cysteamine dose on the renal tissue levels of hydrogen peroxide and nitrite is a paradox. The contradicting grading dose-dependent effects of cysteamine on the renal tissue of IRI rats agree with the studies of Jeitner and Lawrence (2001); Guha et al. (2019). Based on these preclinical studies, a concentration of 10-100 µm improved multiple RC complex disease FBXL4 human fibroblast survival, and protected both complex I (rotenone) and complex IV (azide) *Danio rerio* vertebrate zebrafish disease models from brain death via enhancement of GSH levels, while concentration above this range was linked with cytotoxicity. This cytotoxic effect correlates with a higher concentration of hydrogen peroxide. These pre-clinical studies demonstrated the narrow therapeutic window of cysteamine bitartrate, with toxicity at millimolar levels directly correlating with marked induction of hydrogen peroxide production.

Advance oxidized protein products (AOPPs) are tyrosine-containing protein products with cross-linking peptides that are formed due to the reaction of albumin with hypochlorous acid oxidants from myeloperoxidase-hydrogen peroxide halide system of activated phagocytes (Capeillere-Blandin et al., 2004; Oettl et al., 2008). Many studies have suggested that AOPPs level is elevated during renal dysfunction (Liu et al., 2011) and increased evidence suggests that AOPPs play a vital role in the progression of chronic kidney disease (Li et al., 2007). Due to their role in oxidative stress and inflammation, AOPPs have been reported to induce podocyte apoptosis, and renal tubular lumen epithelial cell injury (Liu, 2009; Iwao et al., 2008). In this study, enhanced levels of tissue free radicals and serum pro-inflammatory cytokines in IRI rats were associated with elevated serum levels of AOPPs. This result agrees with a previous report suggesting that AOPPs are oxidative stress and inflammation biomarkers during renal dysfunction (Guo et al., 2008). Significant reduction in the serum level of AOPPs in cysteamine treatment IRI rats supports a study conducted by Okamura et al., 2014b, which revealed modulatory effects of cysteamine in chronic kidney diseases via inhibition of oxidative stress and myofibroblast activity.

Infiltration of injured tissue with neutrophils and macrophages is a feature of reperfusion injury. The injured tissue is known to release acute pro-inflammatory chemokines like TNF-α and IL-1β (Guillermo and Albert, 2014). These chemical substances can increase vascular permeability, attract leucocytes increase myeloperoxidase (MPO) levels, increase ROS production, activate IL-6 and CRP synthesis from the liver, and even activate cell death receptors (Griffin et al., 2012). In addition, an acute inflammatory response is also associated with the activation of a pro-inflammatory transcription factor called p65NF-kB. This transcription factor is activated by cytosolic stress-associated factors like ROS, TNF-α, hypoxia, and hyperoxia (Chenguang et al., 2002). Upon activation by phosphorylation and ubiquitination by IKB (Inhibitory Kinase B) which also depends on IKK (Inhibitory kinase B Kinase) for phosphorylation, p65NF-kB will move to the DNA promoter region, binds to the response element along with co-transcription associated protein such as histone-acetyl-transferase and methyl transferase to promote the transcription of cyclo-oxygenases and pro-inflammatory cytokines (Alabi et al., 2020). Activation of IL-6 can stimulate the acquired immune system by initiating the differentiation of B-lymphoid cells to immunoglobulins and stimulating cytotoxic T-cells. The pro-inflammatory response can lead to tissue rejection after a kidney transplant. Administration of cysteamine to rats before inducing renal reperfusion injury in this study seems to suppress the release of pro-inflammatory mediators like TNF-α, IL-1β, and MPO and reduced renal expression of p65NFkB. Without treatment of rats with cysteamine, IRI caused a severe increase in the serum level of these mediators and increased renal tissue expression of the pro-inflammatory transcription factor. Considering cysteamine’s activities against pro-inflammatory mediators and p65NFkB, it is possible to describe cysteamine as a potential drug target against acute/delayed tissue graft rejection after a kidney transplant.

Increased production of reactive oxygen and nitrogen species during reperfusion injury is known to cause damage to intracellular organelles, DNA, and mitochondrial membranes (Luciani et al., 2013). DNA damage and stress to the mitochondria membrane are potent stimuli of apoptosis through the classical/intrinsic pathway (Elmore, 2007). Meanwhile, TNF-α can activate cell death receptors to activate the extrinsic apoptosis pathway (Pasparakis and Vandenabeele, 2015). For the intrinsic pathway, intracellular stress will disrupt mitochondrial membrane permeability and activate Bax, which promotes the release of cytochrome C and apoptotic activating factor-1 (Apaf1) referred to as apoptosome. Apoptosomes can now activate caspase 9, an enzyme that will further activate caspase 3 (Redza-Dutordoir and Averill-Bates, 2016). In addition, TNF-α can also activate caspase three via the caspase 8 and 10 extrinsic pathways. In this study, treatment of rats with varying cysteamine doses before inducing renal IRI decreased the tissue expression of caspase 3, a reversal of what we observed in IRI rats without treatment. These results support previous studies on the anti-apoptotic effect of cysteamine (Alabi et al., 2022; Liu et al., 2019).

Creatinine is an important biomarker of renal failure and its clearance depends on the glomerular filtration rate (GFR) (Price and Finney, 2000). From the result obtained in the IRI group, elevated serum levels of creatinine suggest that renal reperfusion injury can reduce the GFR even though the increase was not significant. Although there was an increase in the creatinine level of IRI rats, this result was not significant. The possible explanation for the insignificant effect could be that contralateral kidney compensates for the dysfunction right kidney. The role of the contralateral kidney during unilateral renal IRI was described further by Holderied et al. (2020). The attenuation of creatinine level by 50 mg/kg cysteamine of pretreatment IRI rats supports a previous study of Zurovsky, et al., 1995. This study revealed a significant increase in creatinine level of unilateral renal reperfusion rats and reversed the high level of this renal injury biomarker with antioxidant treatment. A significant decrease in serum creatinine levels of cysteamine-treated IRI rats further supports the protective role of cysteamine against renal reperfusion injury. Also in this study, the evaluation of renal histology of rats in the IRI group showed glomeruli with congested capillaries, and interstitial spaces showed severe hemorrhage. Histological assessment of renal tissue sections of cysteamine-treated rats revealed normal glomeruli, normal mesangial cells, and interstitial space appear normal.

## Conclusion

Based on its activity against major pathways of reperfusion injury such as inflammation, apoptosis, and free radical-induced stress, cysteamine has great potential to be used as a kidney transplant pre-operative drug to prevent renal reperfusion injury.

### Limitation of study

We must notify the specific limitations of the present study; There is a need to run a negative immunohistochemistry control (blank) using tissue and secondary antibody alone. A tunnel assay (terminal deoxynucleotidyl transferase dUTP nick end labeling) is needed to validate DNA damage further. There is also a need to study the role of cysteamine on bilateral renal reperfusion injury.

## Data Availability

The original contributions presented in the study are included in the article/supplementary material, further inquiries can be directed to the corresponding author.

## Abbreviations

MDA, Malondialdehyde; GSH, Reduced glutathione; GPx, Glutathione peroxidase; H_2_O_2,_ hydrogen peroxide; GST, Glutathione-S-Transfease; MPO, Myeloperoxidase; TNF- α, Tumor necrotic factor- α; IRI, Intestinal reperfusion injury; AOPP, Advance oxidized protein product; NF-kB, Nuclear factor Kappa B; IL-1β, Interleukin one beta.
